# Optimal Control of Saccades by Spatial-Temporal Activity Patterns in the Monkey Superior Colliculus

**DOI:** 10.1371/journal.pcbi.1002508

**Published:** 2012-05-17

**Authors:** H. H. L. M. Goossens, A. J. van Opstal

**Affiliations:** Radboud University Nijmegen Medical Centre, Donders Institute for Brain, Cognition, and Behaviour, Department of Cognitive Neuroscience, Section Biophysics, Nijmegen, The Netherlands; New York University, United States of America

## Abstract

A major challenge in computational neurobiology is to understand how populations of noisy, broadly-tuned neurons produce accurate goal-directed actions such as saccades. Saccades are high-velocity eye movements that have stereotyped, nonlinear kinematics; their duration increases with amplitude, while peak eye-velocity saturates for large saccades. Recent theories suggest that these characteristics reflect a deliberate strategy that optimizes a speed-accuracy tradeoff in the presence of signal-dependent noise in the neural control signals. Here we argue that the midbrain superior colliculus (SC), a key sensorimotor interface that contains a topographically-organized map of saccade vectors, is in an ideal position to implement such an optimization principle. Most models attribute the nonlinear saccade kinematics to saturation in the brainstem pulse generator downstream from the SC. However, there is little data to support this assumption. We now present new neurophysiological evidence for an alternative scheme, which proposes that these properties reside in the spatial-temporal dynamics of SC activity. As predicted by this scheme, we found a remarkably systematic organization in the burst properties of saccade-related neurons along the rostral-to-caudal (i.e., amplitude-coding) dimension of the SC motor map: peak firing-rates systematically decrease for cells encoding larger saccades, while burst durations and skewness increase, suggesting that this spatial gradient underlies the increase in duration and skewness of the eye velocity profiles with amplitude. We also show that all neurons in the recruited population synchronize their burst profiles, indicating that the burst-timing of each cell is determined by the planned saccade vector in which it participates, rather than by its anatomical location. Together with the observation that saccade-related SC cells indeed show signal-dependent noise, this precisely tuned organization of SC burst activity strongly supports the notion of an optimal motor-control principle embedded in the SC motor map as it fully accounts for the straight trajectories and kinematic nonlinearity of saccades.

## Author Summary

As the fovea is the only spot on the retina with high spatial resolution, primates need to move their eyes to peripheral targets for detailed inspection. Saccades are the fastest movements of the body, and theoretical studies suggest that their trajectories are optimized to bring the fovea as fast and accurately as possible on target. Speed-accuracy optimization principles explain the stereotyped nonlinear ‘main-sequence’ relationship between saccade amplitude, duration, and peak velocity. Earlier models attributed these kinematic properties to nonlinear neural circuitry in the brainstem but this creates problems for oblique saccades. Here, we demonstrate how the brainstem can be linear, and how instead the midbrain superior colliculus (SC) could optimize saccadic speed-accuracy tradeoff. Each saccade involves the recruitment of a large population of SC neurons. We show that peak firing-rate and burst shape of the recruited cells systematically vary with their location in the SC, and that burst shapes nicely match the eye-velocity profiles. This organization of burst properties fully explains the main-sequence. Moreover, all cells synchronize their bursts, thus maximizing the total instantaneous input to the brainstem, and ensuring that oblique saccades have straight trajectories. We thus discovered a sophisticated neural mechanism underlying optimal motor control in the brain.

## Introduction

**Figure 1:**
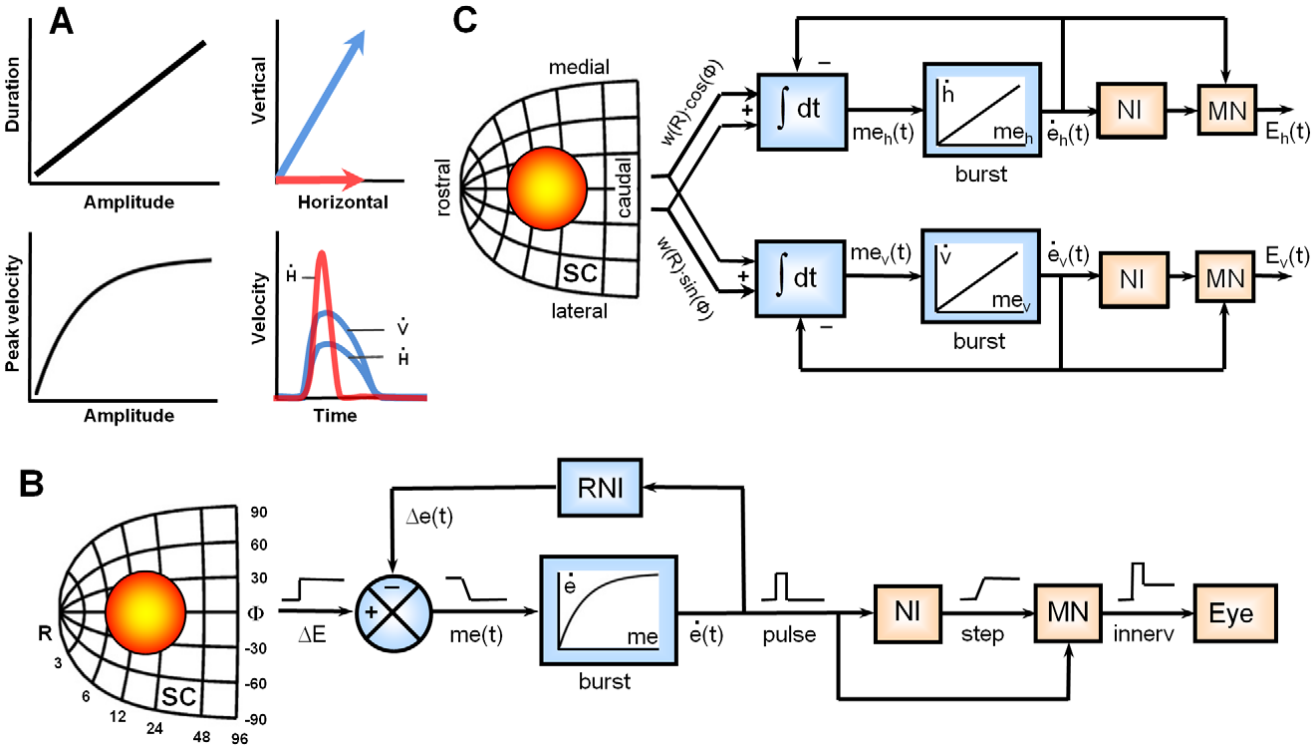
Properties of saccades and models of saccade generation. **A**) Left: main sequence relationship between saccade amplitude, duration and peak velocity. Right: component stretching. Here, an oblique saccade (blue) has very different component amplitudes (top), but horizontal and vertical velocity profiles (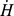 and 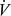) have equal durations and similar shapes (bottom). The horizontal saccade (red) has a much shorter duration and higher velocity than the equally large horizontal component of the oblique saccade. **B**) Classic one-dimensional model which assumes that the superior colliculus (SC) specifies a desired displacement vector (after [12]). Main sequence properties are attributed to a saturating nonlinearity of the burst generator which is controlled through local feedback. Cross-coupling between horizontal and vertical components (not shown) is needed to produce straight saccades. **C**) Linear two-dimensional model of the SC – brainstem saccade generator (after [33]). In this scheme, spatial-temporal activity patterns in the SC specify an intended movement trajectory, which is decoded downstream by ‘spike-vector’ summation: each spike from each neuron adds a site-specific vectorial contribution to movement command. The actual movement is generated by pulse-step activation of the extra-ocular motor neurons, as in B, but the burst generator, which produces the pulse, is linear. ΔE, desired eye displacement; Δ*e*(*t*), current eye displacement; *me*(*t*), dynamic motor error; 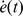, current eye velocity; *w*(*R*), exponential weighting function; *∫dt* temporal integration; Burst, brainstem burst generator; NI, neural eye position integrator; NDI, resettable neural eye displacement integrator; MN, motor neurons; innerv., eye plant pulse-step innervation signal.

Visually evoked saccades have remarkably stereotyped characteristics. Their two-dimensional trajectories are virtually straight, there is a near-linear relationship between movement duration and saccade amplitude, and peak eye velocity versus saccade amplitude follows a nonlinear, saturating relationship (Fig. 1A). These kinematic relations, known as the ‘main sequence’ [1], could indicate a *nonlinearity* in the saccadic system, because for a linear system, when driven by a step input, velocity profiles of all saccades would be self-similar, scaled (by amplitude) versions of each other (see [2] for a discussion of the step-input assumption). As a result, the movement duration would be constant for all saccades, and peak eye velocity would increase linearly with amplitude [3].

Why saccades have these stereotyped kinematics is unknown. Interestingly, theoretical studies [4]–[8] have suggested that the main sequence of saccades could reflect an optimal control strategy, as the system has to cope with several conflicting constraints. More specifically, the properties of internal noise within the system (assumed to increase with activity levels), a low spatial resolution in the peripheral retina, and a penalty for overshooting the target (as corrective commands then have to cross hemispheres), require a speed-accuracy tradeoff. These studies indicated that the optimal trajectories to satisfy such constraints are met by the main-sequence relationships. However, the neural mechanisms for implementing the main-sequence relations are unknown.

Almost every neural model of the saccadic system assumes that the main sequence results from a local feedback circuit in the brainstem [9]–[13] (Fig. 1B). The classic theory is that this circuit receives a step input from the midbrain superior colliculus (SC) encoding the desired eye displacement, and that medium-lead burst cells in the pons are driven by a dynamic motor-error signal which reflects the difference between the desired and the current eye displacement. The pontine burst cells transform this signal into an eye-velocity output, a process known as pulse generation. Most saccade models assume that (due to saturation of peak firing-rates, or neural fatigue) the input-output characteristic of the pulse generator is a saturating nonlinearity that causes the amplitude – peak velocity relation [11]–[15]. While there is compelling evidence that the firing-rate of these neurons encodes eye velocity [11], [16]–[19], there is surprisingly little data to support the assumption that their input-output characteristic underlies the nonlinear main sequence. A critical problem is that the true nature and dynamics of their input signals are unknown. It is also not clear how a saturating nonlinearity in the horizontal and vertical brainstem circuits could support optimal control in two dimensions. Because the generation of straight saccades in oblique directions involves stretching the horizontal and vertical velocity components in such a way that they are scaled versions of each other (Fig. 1A), an intricate cross-coupling between the horizontal and vertical pulse generators would be required [20]–[25]. Clearly, straight trajectories are optimal in the sense that they constitute the shortest path to the endpoint.

Here, we study the role of the superior colliculus in the optimal control of saccades. The deeper layers of the SC form a topographic map of saccade vectors (Figs. 1B and 1C), which is organized in eye-centered coordinates [26]. Neurons in this motor map fire a brisk burst of action potentials tightly coupled to the onset and duration of the saccade. Cells near the rostral pole of the SC are involved in the generation of small saccades, while cells at caudal sites encode large eye movements. Saccade direction in the contralateral hemifield is represented along the medial-lateral dimension. The range of movement vectors for which an SC neuron is recruited is called its movement field [27], [28], and it has been inferred from the size and shape of the cells' movement fields that each saccade is associated with a location-invariant two-dimensional Gaussian activation profile in the map [28]. However, how the temporal dynamics of activity within the active population contributes to the movement kinematics is not clear [29]–[33].

Because the SC is an important sensorimotor interface in the control of saccade behavior [34]–[36], it could be in an ideal position to optimize speed-accuracy tradeoff. In line with this notion, we recently found that the nonlinear kinematics of saccades are already embedded in the spatial-temporal SC activity patterns [33]. More specifically, we demonstrated that measured SC firing patterns, when used to drive a linear feedback model of the brainstem, produce realistic, straight saccades that obey the nonlinear main sequence. As shown in Fig. 1C, this analysis assumed that i) the spatial-temporal activity patterns in the SC motor map are decoded by a linear ‘spike-vector’ summation mechanism in which each spike from each neuron adds an independent, site-specific vectorial contribution to the saccade command, and that ii) this command is executed by two independent, linear feedback circuits in the brainstem that control the horizontal and vertical movement components, respectively. Thus, none of the nonlinear properties of saccades (component stretching, skewness of velocity profiles, and main-sequence relations) were built into the model; they all emerged from the measured SC activity patterns (see also Video S1).

However, the mechanism by which the recruited SC population generates these properties remained unclear. In a recent theoretical study we proposed that a possible mechanism could reside in a topographic organization of burst properties of saccade-related SC cells [37]. This theory predicts that the SC could specify the nonlinear main sequence of saccades if the saccade-related bursts vary from brief and intense in the rostral zone, to less intense and of longer duration in the caudal zone, while keeping the total number of spikes constant. It also predicts a systematic rostral-to-caudal increase in burst skewness, because skewness of the eye-velocity profiles increases with saccade amplitude [38]. Moreover, if the SC indeed acts as an optimal controller, one would expect that cells in the recruited population synchronize their burst profiles because this would ensure that the net SC movement command specifies a straight trajectory at optimal speed. Note, however, that if burst durations and skewness indeed vary along the map, such synchronization of burst activity can only occur if the shape of each cell's burst depends on the planned movement, rather than on its anatomical location in the motor map. We thus predicted that burst shapes are different depending on whether a cell is recruited for small versus large saccades. Finally, optimal control theories predict that speed-accuracy tradeoff is constrained by noise, which increases with the amplitude of the control signals [5]–[7]. This suggests that SC cells might exhibit signal-dependent noise.

Several studies have examined the spatial-temporal organization of saccade-related burst activity in the SC [29]–[33], but a detailed, quantitative analysis is still missing. Furthermore, the possibility that populations of movement cells might encode the optimal kinematics of different movements through a topographic gradient of firing properties has never been studied. In the present paper we therefore characterized the activity patterns of a large population of saccade-related SC neurons, widely distributed across the motor map. Our results reveal a highly systematic organization of burst properties along the rostral-to-caudal extent of the SC motor map, which can fully account for the nonlinear kinematics of saccades, their straight trajectories in oblique directions, and the skewed shape of their velocity profiles. Moreover, we demonstrate signal-dependent noise in the number of spikes of saccade-related bursts, as predicted by optimal-control theories. These remarkable findings strongly support the notion of an optimal motor-control principle embedded in the SC.

## Results

If the SC plays a role in optimal control, and if the brainstem indeed acts as a linear system, one would expect that: 1) individual SC neurons exhibit signal-dependent noise, 2) peak firing-rate, burst duration and burst skewness at the center of the recruited population all depend systematically on its rostral-caudal coordinate in the SC motor map, while the number of spikes in the burst remains fixed, 3) the shape of a neuron's burst depends systematically on the actual movement, and 4) all cells in the recruited population synchronize their burst profiles. In what follows, we present quantitative evidence for each of these predictions.

### Signal-dependent noise

One of the central premises in the optimal control theories proposed by Harris and Wolpert [5] and Tanaka et al. [7] is that the amount of noise in the control signal is proportional to signal amplitude, but experimental evidence for this assumption is still limited [39]–[41]. Here we test whether such signal-dependent noise exists at the level of the SC motor map. Towards that end, we quantified the trial-to-trial variability of saccade-related burst activity of SC neurons for visually guided saccades into their movement field.

**Figure 2:**
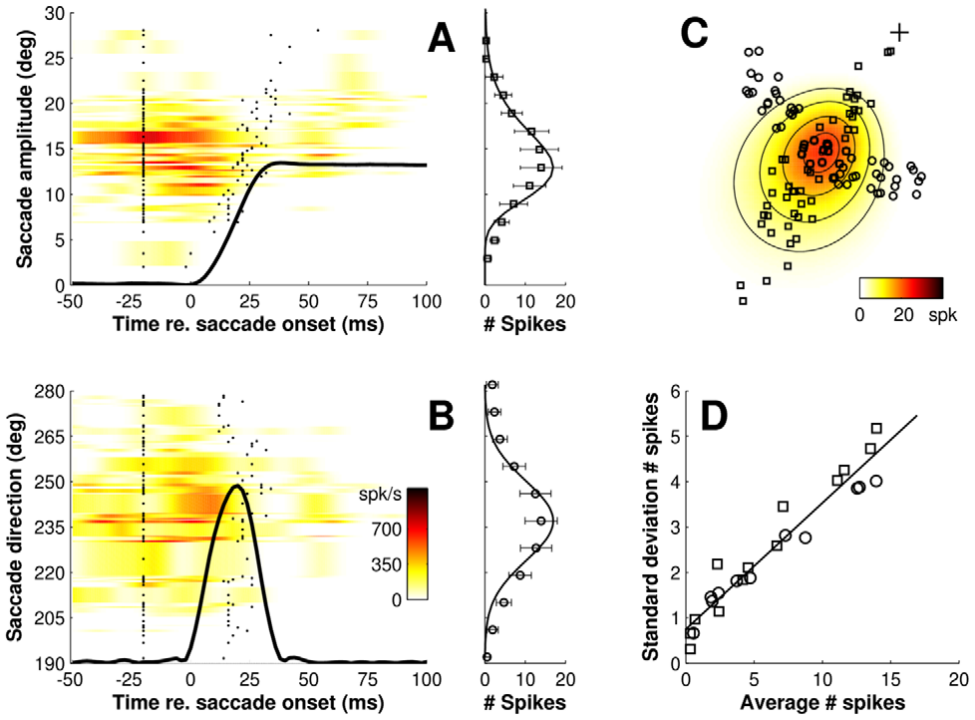
Movement field scan of a saccade-related SC neuron illustrating the presence of signal-dependent noise. **A**) Spike density (color code) as a function of time for saccades in and near the center of the movement field, with trials sorted by amplitude of the movement (amplitude scan; fixed direction *Φ*≅237°). Tick marks indicate spike-count windows (20 ms before onset to 20 ms before offset of the saccade). Superimposed is the average eye position of saccades towards the movement field center. Spike counts (open symbols) are displayed as running averages across 6° wide bins. Error bars indicate the trial-to-trial variability in spike counts (±1 SD). **B**) Same for a direction scan through the center of the movement field (fixed amplitude *R*≅13°). Spike counts were averaged across 13° wide bins. Average velocity profile of saccades towards the center is superimposed. **C**) Spatial extent of the movement field together with the endpoints of saccades (re. to initial fixation, +) included in the amplitude and direction scan. Color code: movement field description (Methods, Eq. 3) of the number of spikes in the burst as function of saccade amplitude and direction. Contour lines are drawn at [0.5, 1.0, 1.5 and 2.0]⋅σ_mf_. **D**) Spike-count standard deviations as a function of mean number of spikes in the burst. Linear regression line (solid) was calculated from the pooled data of the amplitude (squares) and direction (circles) scans.

Figure 2 illustrates the results for a typical cell. The left-hand panels show the instantaneous firing rate of the cell (color code) as a function of time for selected saccades of different amplitudes (Fig. 2A, ‘amplitude scan’) and directions (Fig. 2B, ‘direction scan’). Individual trials are sorted according to saccade amplitude and direction, respectively. For each saccade, the burst magnitude was quantified from the raw data by counting all spikes in the time window between the two tick marks (identifying saccade onset an offset with a 20 ms lead time), and the resulting spike counts are displayed in the adjacent panels as running averages. Saccade endpoints of the responses in Figs. 2A and B (squares and circles, respectively) are plotted in Fig. 2C, together with a two-dimensional representation of the cell's movement field (Methods). This movement-field plot shows that the number of spikes in the burst (color code) varies systematically with saccade amplitude and direction. However, as can be seen in Figs. 2A and B, it is not only the mean number of spikes in the burst (open symbols) that depends systematically on the amplitude and direction of the saccade vector; also the trial-to-trial variability in the spike counts changes systematically (error bars indicate ±1 SD). More specifically, when the spike-count variability is plotted as a function of the average number of spikes in the burst (Fig. 2D), it appears for this cell that the standard deviation increases almost linearly with the mean. To quantify this relation, we fitted a regression line to the data. Note that the intercept is practically zero. The slope of the regression line thus provides a good measure of the so-called coefficient of variation, which is the ratio between the standard deviation and the mean (Methods). For the neuron in Fig. 2, the coefficient of variation was *C_v_* = 0.278±0.016 (mean±SEM).

**Figure 3:**
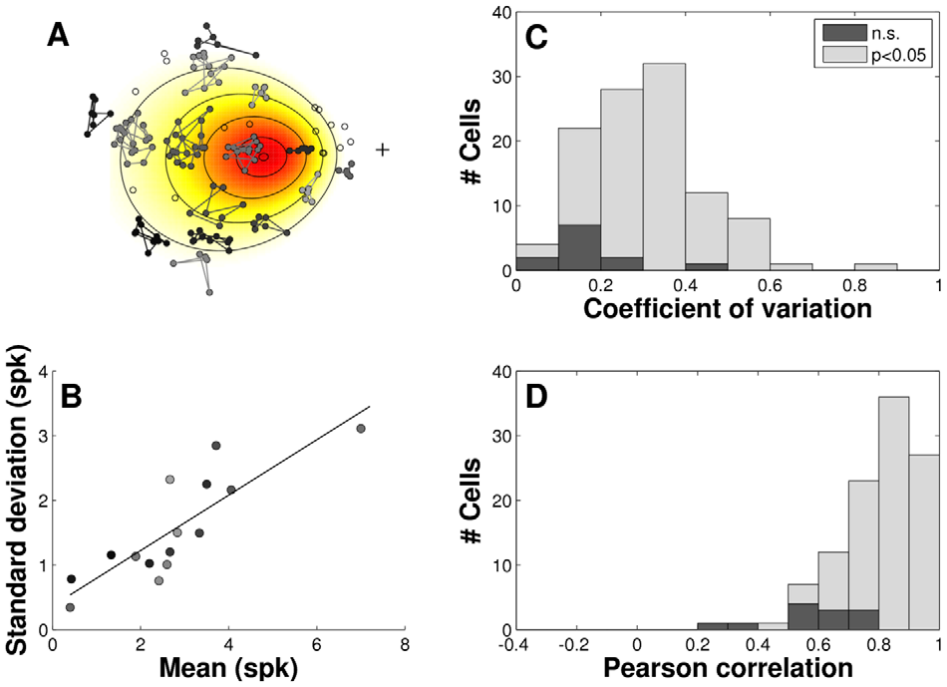
Signal-dependent noise across the population of recorded SC neurons. **A**) Movement field data from an individual neuron was parsed into clusters (here, n = 15) containing at least 5 saccades to nearby locations. Preferred vector of the cell shown here was [*R*,Φ] = [12,183]°. Endpoints of saccades belonging to the same cluster are gray-coded and connected by line segments. **B**) For each cluster, mean and standard deviation of the number of spikes in the burst were computed (Methods). The relation between these variables is characterized by the coefficient of variance, *C_v_*, which we determined from the slope of the linear regression line (solid, *C_v_* = 0.43±0.08). **C**) Stacked histograms show the distribution of *C_v_* for the population of cells in which the regression was statistically significant (bright; n = 96) and for the population of cells for which it was not significant (dark; n = 12). Legend: p-value of the regression (two-tailed *t*-test); n.s., not significant. **D**) Same for the distribution of linear correlation coefficients.

To quantify the signal-dependent variability of saccade-related bursts for the entire population of cells, we selected for each SC neuron a series of non-overlapping clusters of closely matched saccade responses. This analysis, which is illustrated in Fig. 3A and B, typically included the entire movement field. Figures 3C and D summarize the results for all 108 cells for which we obtained sufficient data (see Methods, for details). Note that for the vast majority of cells (89%, n = 96) spike-count variability increased significantly with the mean number of spikes (two-tailed t-test, p<0.05). The mean (±SEM) value of the coefficient of variation across the cell population was 0.306±0.014. These findings thus indicate that motor commands generated by individual SC neurons are endowed with a considerable amount of signal-dependent noise.

Because signal-dependent noise alone cannot fully account for the variability in observed saccade trajectories, it has been suggested that cell activity may also be endowed with signal-independent noise [42]. We therefore quantified the level of signal-independent noise in the SC by analyzing the intercepts of the regression lines (c.f., Fig. 2D and Fig. 3B). Averaged across our sample of cells, the mean intercept was indeed significantly different from zero (two-tailed t-test, p<0.001), but with an average value of 0.54±0.03 spikes (mean±SEM) it reached only 3.0±0.2% of the cells' average peak response.

### Spatial variation of burst properties

As mentioned in the introduction, we recently proposed on the basis of model simulations that a topographic organization of burst properties within the SC motor map could underlie the nonlinear kinematics of saccades [37]. More specifically, our simulations showed that the SC population could specify the main sequence if the saccade-related bursts vary from brief and intense in the rostral zone (small-saccade area), to less intense and of longer duration in the caudal zone (encoding large saccades) while keeping the number of spikes constant.

**Figure 4:**
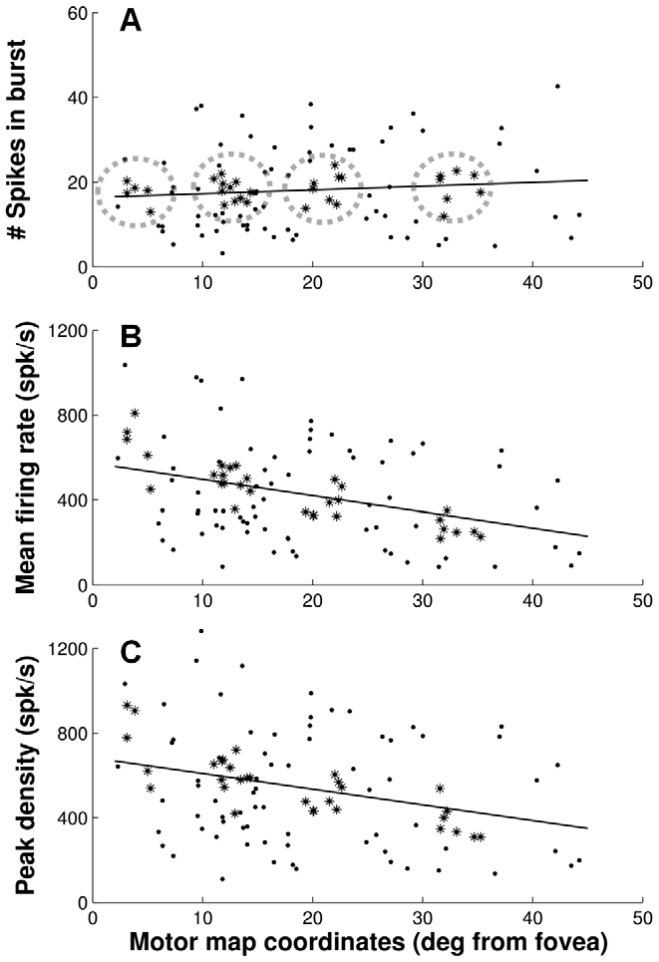
Burst magnitude across the SC motor map. Three different activity measures were used to quantify the magnitude of the saccade-related bursts of SC neurons for movements towards the center of their movement field as a function of their rostral-to-caudal location in the SC motor map: **A**) number of spikes in the burst, **B**) mean firing rate and **C**) peak spike density. Cells were selected for having at least five saccades into the center of their movement field (within 0.5⋅σ_mf_). Highlighted cells (asterisks) in four clusters of cells (dashed circles) were selected for producing ∼18 spikes for their preferred saccade (c.f., Fig. 5). Linear regression lines (solid) were fitted to the data from all n = 103 cells.

To test these theoretical predictions, Fig. 4 quantifies several burst properties of saccade-related SC cells for saccades towards the center of their movement field as a function their anatomical rostral-to-caudal location in the motor map: number of spikes in the burst (Fig. 4A), mean firing rate (Fig. 4B), and peak firing rate (Fig. 4C). Spike counts and mean firing rates were calculated from the raw data, while peak firing rates were estimated from the spike-density functions. We indexed the rostral-to-caudal location of each cell in the motor map by the amplitude of its preferred vector. Cells (n = 103) were selected for having at least 5 saccades into the center of their movement field (Methods).

Note that the spike counts are remarkably constant across the rostral-to-caudal extent of the SC as cells at each location fire on average about 18 spikes for their preferred saccade. Mean and peak firing rates, on the other hand, decrease systematically with the rostral-to-caudal location of the cells. To quantify these topographic relations we fitted linear regression lines (solid) to each of the three datasets. The numerical results are listed in Table 1 (top entries). This regression analysis showed there is no significant correlation between spike counts and preferred amplitudes, and thus anatomical location, of the neurons (Pearson correlation, p>0.3). Correlations between firing rates and preferred amplitudes, however, were significantly different from zero (t-test, p<0.005). Slopes of the regression lines (mean±SEM) in Fig. 4B and C were −7.6±1.9 and −7.3±2.3 spk/s per deg, respectively. The data thus indicate an almost two-fold decrease in firing rates from small to large saccades within the oculomotor range.

**Table 1:** Summary of topographic variations in burst parameters.

	*Sigma*	*Mean*	*Slope*	*Intercept*	*Correlation*
***#Spikes in burst, N_s_***	-	18±9	0.09±0.08	16±1.8	0.104^†^
		(spk)	(spk ⋅ deg^−1^)	(spk)	
***Mean firing rate, F_m_***	-	429±216	−7.6±1.9	573±42	−0.364**
		(spk/s)	(spk/s ⋅ deg^−1^)	(spk/s)	
***Peak density, F_p_***	2	632±266	−7.5±2.5	774±53	−0.288*
	5	545±252	−7.3±2.3	683±50	−0.300*
	10	480±225	−5.8±2.1	589±45	−0.265*
	(ms)	(spk/s)	(spk/s ⋅ deg^−1^)	(spk/s)	
***Time to peak, T_p_***	2	−1.7±9	−0.05±0.09	−0.9±1.9	−0.052^†^
	5	−1.1±8	−0.02±0.08	−0.7±1.7	−0.025^†^
	10	−2.0±7	−0.02±0.07	−1.7±1.6	−0.026^†^
	(ms)	(ms)	(ms ⋅ deg^−1^)	(ms)	
***Skewness, γ_1_***	-	0.11±0.23	0.010±0.002	−0.09±0.04	0.460***
			(deg^−1^)		
***Coeff of variation, Cv***	-	0.28±0.16	0.000±0.002	0.28±0.03	−0.006^†^
			(deg^−1^)		

*Sigma*: width σ of the Gaussian smoothing kernel used to compute the spike density functions from which *F_p_* and *T_p_* were estimated. Not applicable for *N_s_*, *F_m_*, *γ_1_* and *C_v_* because they were calculated from the raw spike data. *Mean*: parameter values (mean ± SD) averaged across all n = 103 cells included in this analysis with the corresponding standard deviation. *Slope and intercept*: linear regression coefficients (mean ± SEM) that quantify changes in burst parameters as a function of the rostral-to-caudal location of cells in the SC motor map (c.f., Figs. 4 and 6). *Correlation*: Pearson's correlation coefficient (two-side t-test: *, p<0.01; **, p<0.001; ***, p<0.000001; †, not significant, p>0.1). Since results were consistent across animals, listed values are from analyses performed on the pooled data from four animals.

Because mean firing-rates were computed from the number of spikes divided by saccade duration (Methods), a reduction in mean firing-rate with amplitude could, in principle, result from the confounding factor that saccade duration increases linearly with amplitude (c.f., Fig. 1A). However, this potential confound does not play a role for the peak firing-rate. In our analysis, the absolute peak firing-rate values are partly determined by the width of the Gaussian smoothing kernel (see Table 1 for results with different values of 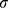), but for a fixed kernel width (here, 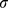 = 5 ms), the changes in peak firing-rate across the population of cells are entirely due to changes in the temporal distribution of spikes within their bursts. The data in Fig. 4C thus confirm the presence of a real rostral-to-caudal gradient in the cells' activity patterns, which – to our knowledge – has not been documented before.

**Figure 5 pcbi-1002508-g005:**
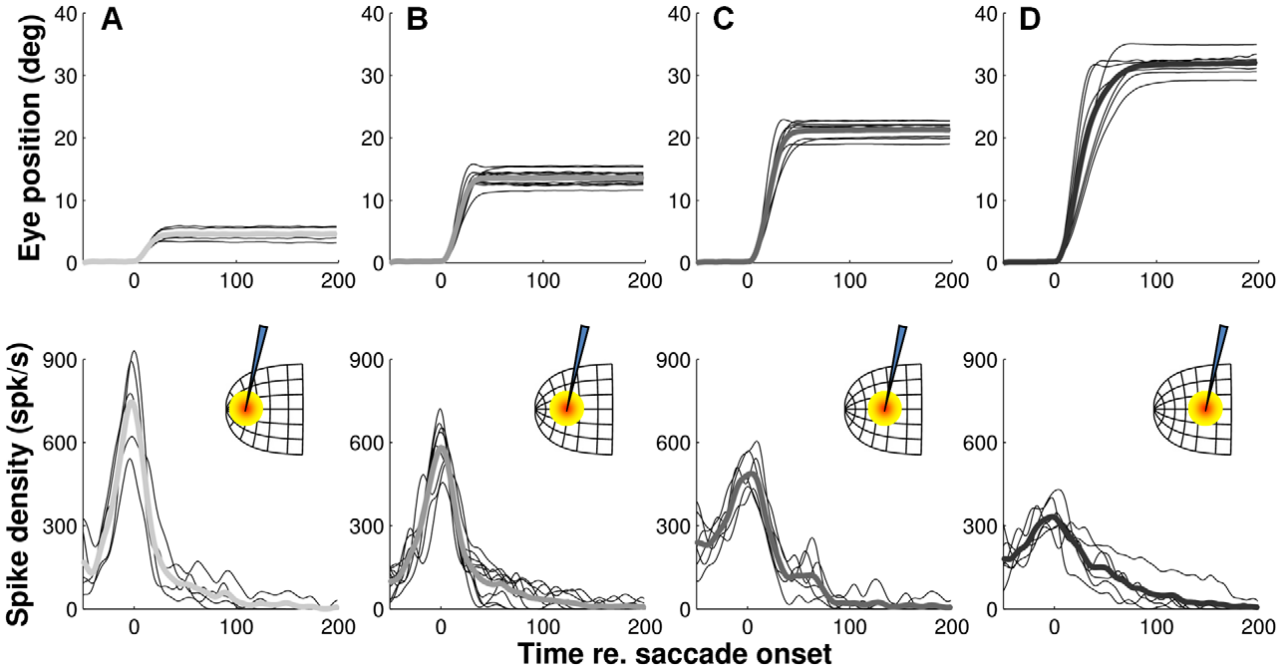
Systematic rostral-to-caudal changes in temporal burst profiles. **A–D**) Illustrated are the temporal firing patterns of saccade-related SC neurons at four different rostral-to-caudal locations in the motor map (insets). Movements (top) corresponded with their preferred vector. Eccentricities of the cells (as indexed by their preferred vectors) were about 5°, 13°, 21° and 32°, respectively. All cells (n = 5, n = 11, n = 8, and n = 7, respectively) were selected for producing ∼18 spikes for their preferred saccade (i.e., highlighted cells in Fig. 4). Thin and thick lines are cell and cluster averages, respectively. Importantly, spike density functions were not normalized in any way.

This spatial gradient is further illustrated in Figs. 5A–D which show the full temporal burst profiles of four clusters of cells that fired ∼18 spikes for their preferred saccade (dashed circles in Fig. 4A). Insets illustrate, schematically, the location of the recording sites in the motor map. It is important to note that this analysis does not involve any normalization of activity. Thin lines represent the mean spike-density functions of the individual cells, where data are aligned with saccade onset and averaged across at least 5 saccades into the center of the movement field (within 0.5⋅σ*_mf_*). Amplitudes of the preferred vectors were about 5°, 13°, 21°, and 32°, respectively, as may be inferred from the corresponding eye-position traces. Thick lines are the grand averages of the responses. Note that the systematic rostral-to-caudal changes in mean and peak firing rates are quite obvious from these discharge profiles too. Cells recorded in the rostral region of the SC (Fig. 5A) clearly showed much higher peak firing rates and shorter bursts for their preferred vector than the ones in the caudal SC (Fig. 5D), while cells found at intermediate locations (Fig. 5B and 5C) had intermediate firing rates and burst durations.

**Figure 6:**
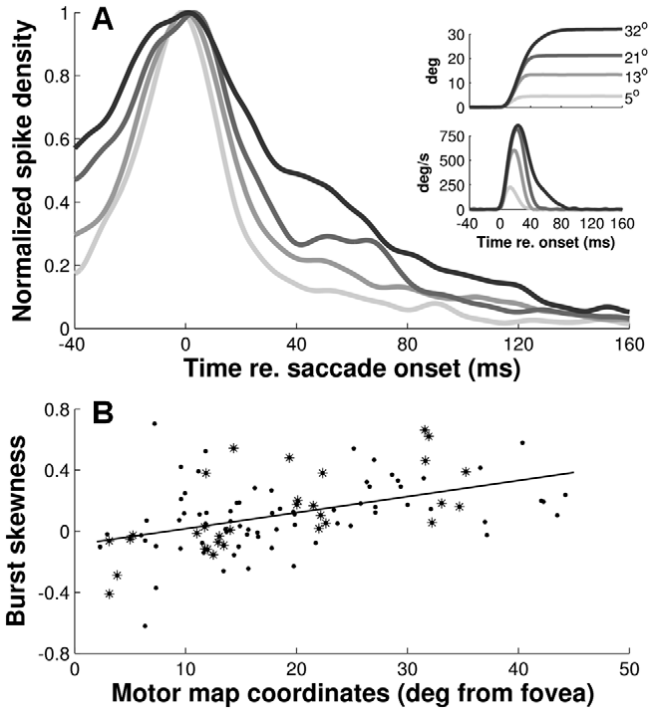
Burst profiles show amplitude-dependent skewness. **A**) Average spike-density profiles of the four clusters of cells normalized with respect to their peaks. Cells in each cluster were selected for producing ∼18 spikes for their preferred saccade (c.f., Figs. 4 and 5). Insets: average eye position traces and eye velocity profiles for the each of the four cell clusters. **B**) Skewness of saccade-related bursts for preferred saccades as a function motor map coordinates. The relationship was quantified with linear regression (solid).

In Fig. 6A we normalized the average burst profiles from the four clusters of cells (Figs. 5A–D) with respect to their peak. The top-right insets show the main sequence behavior and velocity profile skewness [38] of the corresponding average eye movements (data replotted from Fig. 5). This analysis shows that the peak firing rate occurs at about the same instant relative to saccade onset (see Table 1, for further quantification) while the burst duration increases systematically with the preferred amplitude of the cells (indexed by the gray-scaling). Hence, these results indicate that, just like the saccade velocity profiles, the skewness of the bursts increases systematically with saccade amplitude (and duration).

Note, however, that the shapes of the spike-density waveforms in Fig. 6A also depend on the width of the Gaussian smoothing kernel that is used to compute the instantaneous firing rates (here, 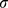 = 5 ms). To circumvent this problem, in Fig. 6B, we therefore calculated burst skewness directly from the distribution of spike moments in the burst (Methods), rather than from the spike density functions. As in Fig. 4, cells (n = 103) were selected for having at least five saccades into the center of their movement field. Note that bursts associated with small saccades (produced by cells in the rostral region of the SC) are nearly symmetric (skewness about zero) while bursts associated with large saccades (produced by cells in the caudal SC) have longer tails towards the end of the saccade (positive skewness). Linear regression analysis showed a significant, positive correlation (t-test, p≪0.001) between the skewness of the bursts and the preferred amplitudes of the cells. This shows that burst skewness indeed increases systematically with the cells' rostral-to-caudal location in the motor map. Table 1 summarizes the numerical results of this regression analysis (fifth entry).

The systematic increase in burst skewness with saccade amplitude was not related to any change in spike-count variability; the coefficient of variation computed for these bursts (Eq. 4) was more or less constant across the map (Table 1), and did not significantly correlate with burst skewness (t-test, p>0.28).

The topographic variations in burst properties that are revealed by the analyses in Figs. 4–6 were consistent across animals. In all three animals for which we obtained a sufficient number of cells at different locations in the map (n>10; smallest eccentricity <4°; largest eccentricity >40°), we observed a nearly constant number of spikes across the map, a systematic rostral-to-caudal decrease in mean and peak firing rates, and a systematic rostral-to-caudal increase in burst skewness. In the fourth animal, recording sites did not span a large enough range of eccentricities (11°<R<15°) to obtain reliable fit results.

### Burst dynamics depend on the actual saccade vector

So far the analyses quantified the burst properties of SC neurons for saccades towards the center of their movement field, i.e., when the cells are part of the central region of the recruited population. However, SC cells are recruited for a wide range of saccades (the movement field) that all have different kinematics depending on their amplitude. The question therefore arises what happens to the burst of a given cell when it is recruited for saccades that deviate from its preferred vector.

**Figure 7:**
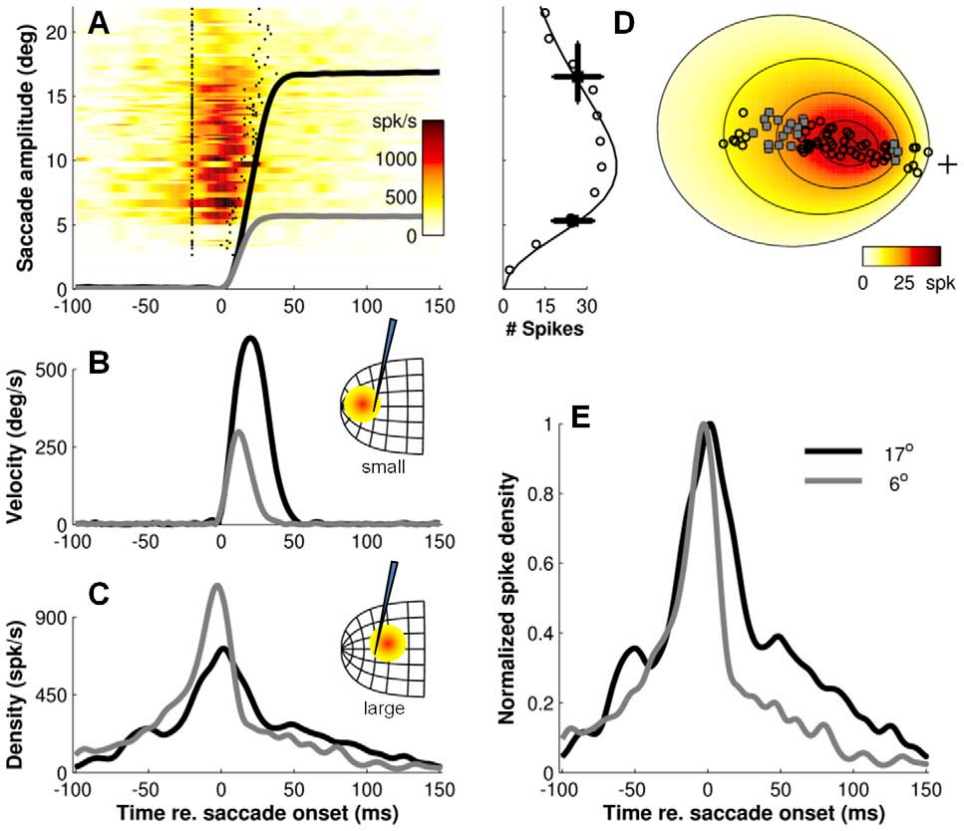
Burst shapes depend on planned movement. Temporal firing patterns of a neuron during small and large saccades for which the cell fired about same number of spikes. **A–C**) Spike density (color code) as a function of time for saccades of different amplitude in the preferred direction of the cell. Data are sorted by saccade amplitude. Tick marks indicate spike-count windows. Gray traces: average eye position, eye velocity and spike density functions for small, 6° saccades (n = 5). Black traces: averaged data for large, 17° saccades (n = 16) for which the cell fired about the same number of spikes (*N_s_*≅25). Inset in A: running average of the number of spikes as a function of saccade amplitude. Vertical line segments: amplitude range of saccades included in the two datasets. Horizontal line segments: mean±SD of the corresponding spike counts. Insets B and C: schematic drawing of population activity relative to the recording site. **D**) Location and extent of the movement field (color code) plus saccade endpoints (symbols). **E**) Normalized spike density functions for the two saccade vectors. Note that the shapes of the bursts for small saccades (light-gray traces) and large saccades (dark-gray traces) are clearly different. Peak firing rates occurred at about the same instant relative to movement onset regardless of the movement amplitude, but burst durations were shorter and peak firing rates were higher when the cell took part in the small saccades than when it participated in the large saccades.

**Figure 8:**
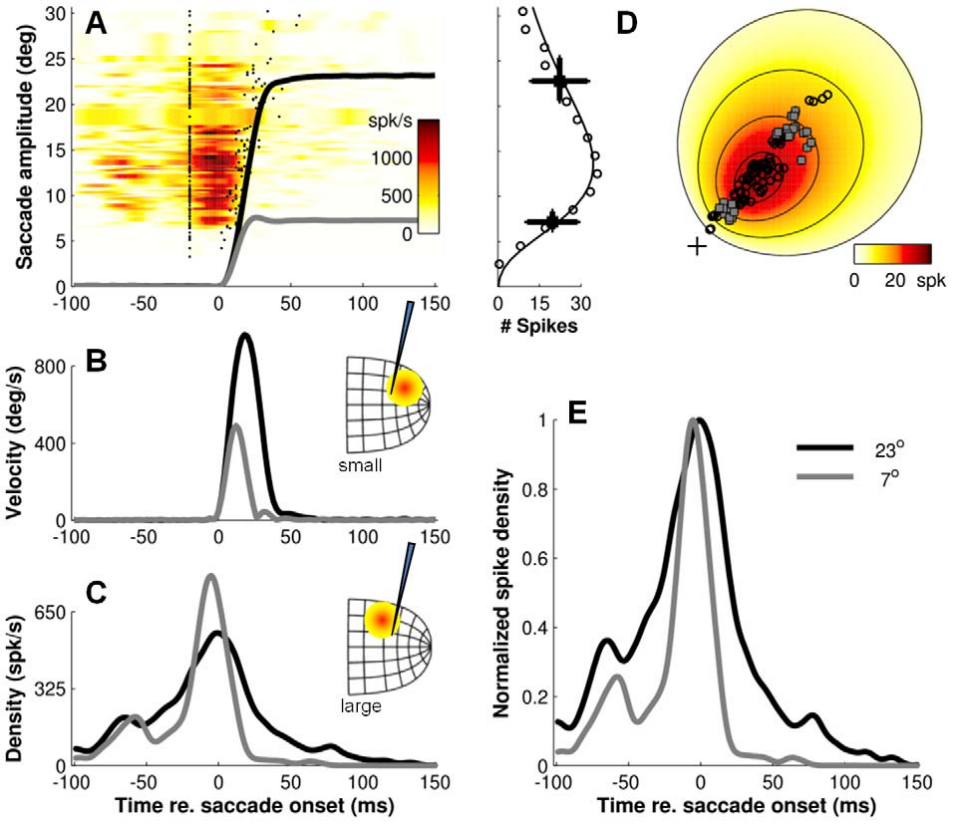
Same analysis of burst activity as in **Fig. 7**, but now applied to data from a different neuron in a different animal. Note different burst profiles for the small (n = 10) versus large (n = 17) saccades for which the cell fired the about same number of spikes (*N_s_*≅20), indicating that the burst parameters depend on the actual saccade in which the neuron is participating, rather than on its topographic location in the motor map.

To address this question, we examined the shape of the burst profiles for saccades towards more eccentric locations within the movement field. More specifically, we compared the burst dynamics when the same cells are part of the rostral region of the recruited population (i.e. they are recruited for saccades that are larger than their preferred movement), with the burst dynamics when they belong to the caudal region of the recruited population (for saccades that are smaller than the preferred one; see motor-map insets Figs. 7 and 8, for schematic illustration).

If saccade-related SC cells indeed participate in specifying the optimal saccade trajectory [33], one would expect that the shape of their bursts is different depending on whether the cells are recruited for saccades that are smaller than their preferred vector, versus whether they are recruited for saccades that are larger than their preferred vector, because the shapes of the velocity profiles for smaller and larger saccades differ (Fig. 6, inset). Alternatively, the shape of the bursts could remain the same regardless of the actual saccade vector. Such behavior is expected if the burst activity of each cell represents only the weight of its preferred vector in a downstream center of gravity computation of the saccade goal [43]–[45]. In this case, the number of spikes produced by each cell is determined by its input, but the temporal firing properties only depend on its location in the motor map.

To dissociate between these two possibilities, Figs. 7 and 8 show the measured burst profiles of two cells in for a series of large and small saccades for which the cell fired the *same* number of spikes. We chose two cells from the central region of the SC (preferred amplitudes 9.5° and 13.5°, respectively) because these cells fired vigorous bursts for saccades with distinctly different kinematics. Note that the shapes of the bursts for small saccades (light-gray traces) and large saccades (dark-gray traces) are clearly different. For both cells, peak firing rates occurred at about the same instant relative to movement onset regardless of the movement amplitude, but burst durations were shorter and peak firing rates were higher when the cell took part in the small saccades than when it participated in the large saccades. These examples thus suggest that the shape parameters of the burst depend systematically on the actual saccade to which the neuron contributes, rather than on its topographic location in the motor map.

### Population dynamics

**Figure 9:**
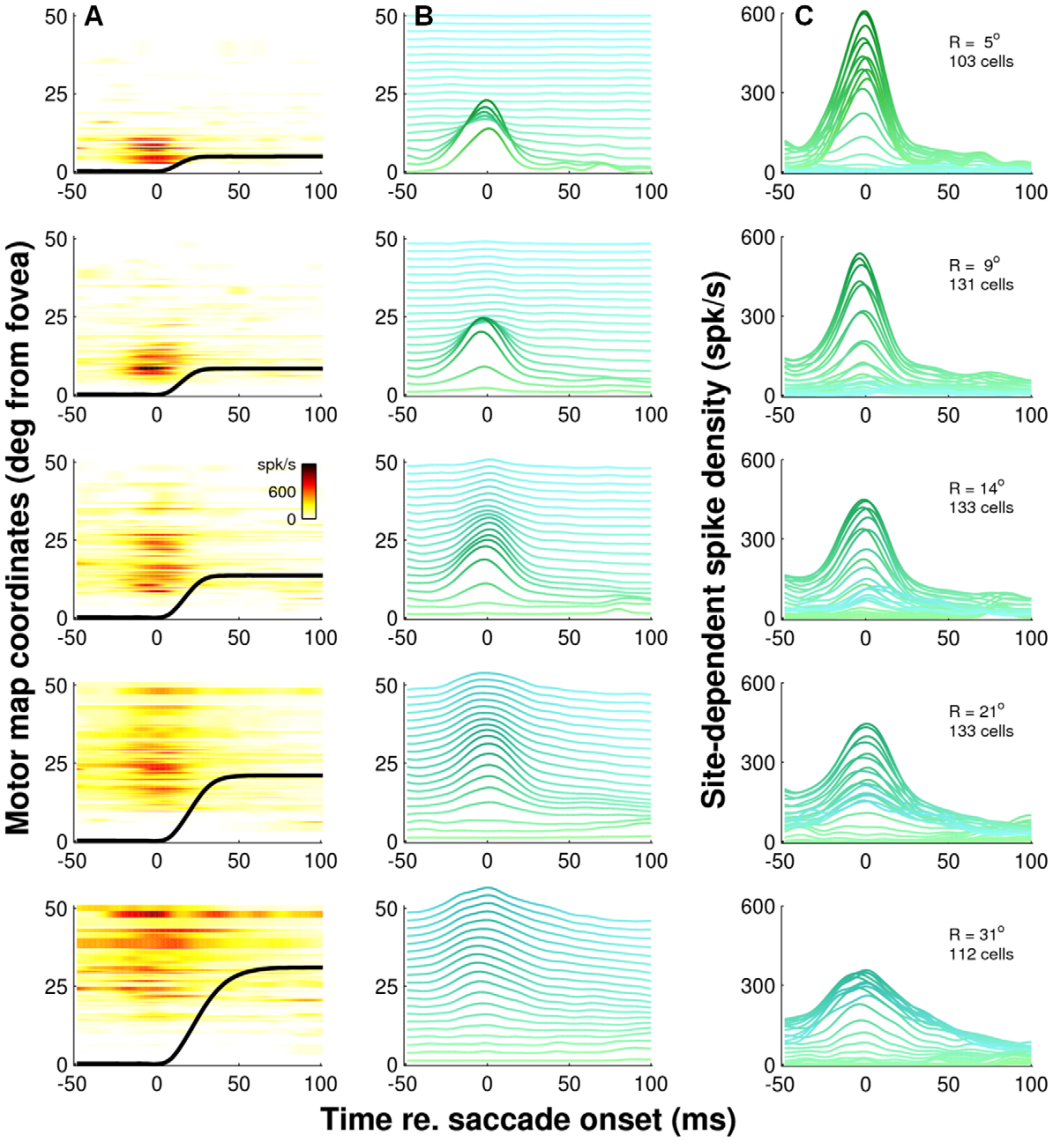
Population dynamics within the motor map. Temporal discharge patterns of saccade-related neurons along the rostral-to-caudal extent of the SC for five different saccade amplitudes. Saccade direction matched the preferred direction of each cell. **A**) Instantaneous firing rates (color code) of individual cells, sorted according to their rostral-to-caudal location in the SC motor map. Average eye position traces are superimposed. **B**) Mean discharge profiles of cells at different locations in the map. Each spike density function is the average activity of cells recorded in restricted region of the SC. Bin centers at 2.5° intervals. Burst profiles are shifted upward and color-coded according to the motor map coordinates. **C**) Site dependent discharge profiles computed at 0.2 mm intervals. As in B, hues of the individual traces refer to the rostral-to-caudal location, with green corresponding to rostral sites and cyan to caudal sites while color saturation is proportional to the instantaneous spike density. Discharge profiles of individual cells are not normalized. Note that for a given saccade amplitude the burst profiles along the rostral-to-caudal extent of the SC appear to have very similar shapes, which change systematically as function of saccade amplitude (and duration).

**Figure 10 pcbi-1002508-g010:**
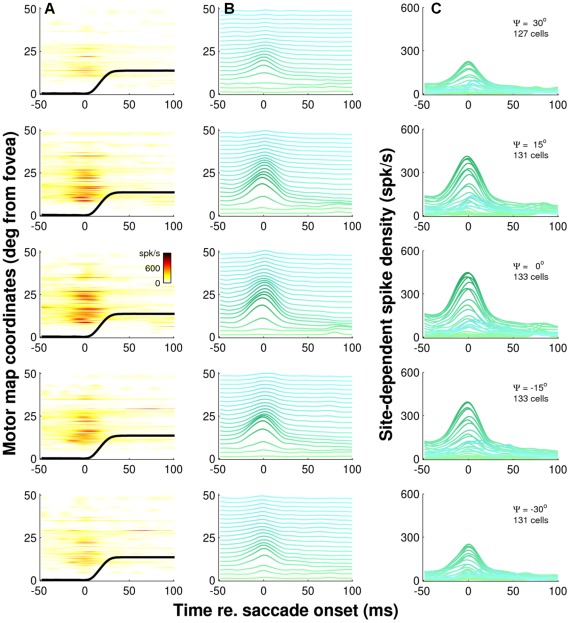
Neurons within the recruited population have similar burst dynamics. Temporal discharge patterns of saccade-related neurons for 14° saccades in five different directions ψ relative to their preferred vector. Positive values of ψ correspond with counter-clockwise rotations. Same layout as Fig. 9 but note that each row now represents the burst profiles of cells along a rostral-to-caudal iso-direction line though the active population. ψ = 0° runs through the center of the population. The other four iso-direction lines show firing patterns at different medial-lateral locations. Note that the peak firing rates decrease systematically with increasing rostral-to-caudal and increasing medial-to-lateral distance from the center of the population activity while the shape of the temporal burst profiles remains remarkably similar. All discharge profiles are approximately scaled versions of each other indicating that the temporal dynamics of burst activity is very similar throughout the population of recruited cells.

**Figure 11:**
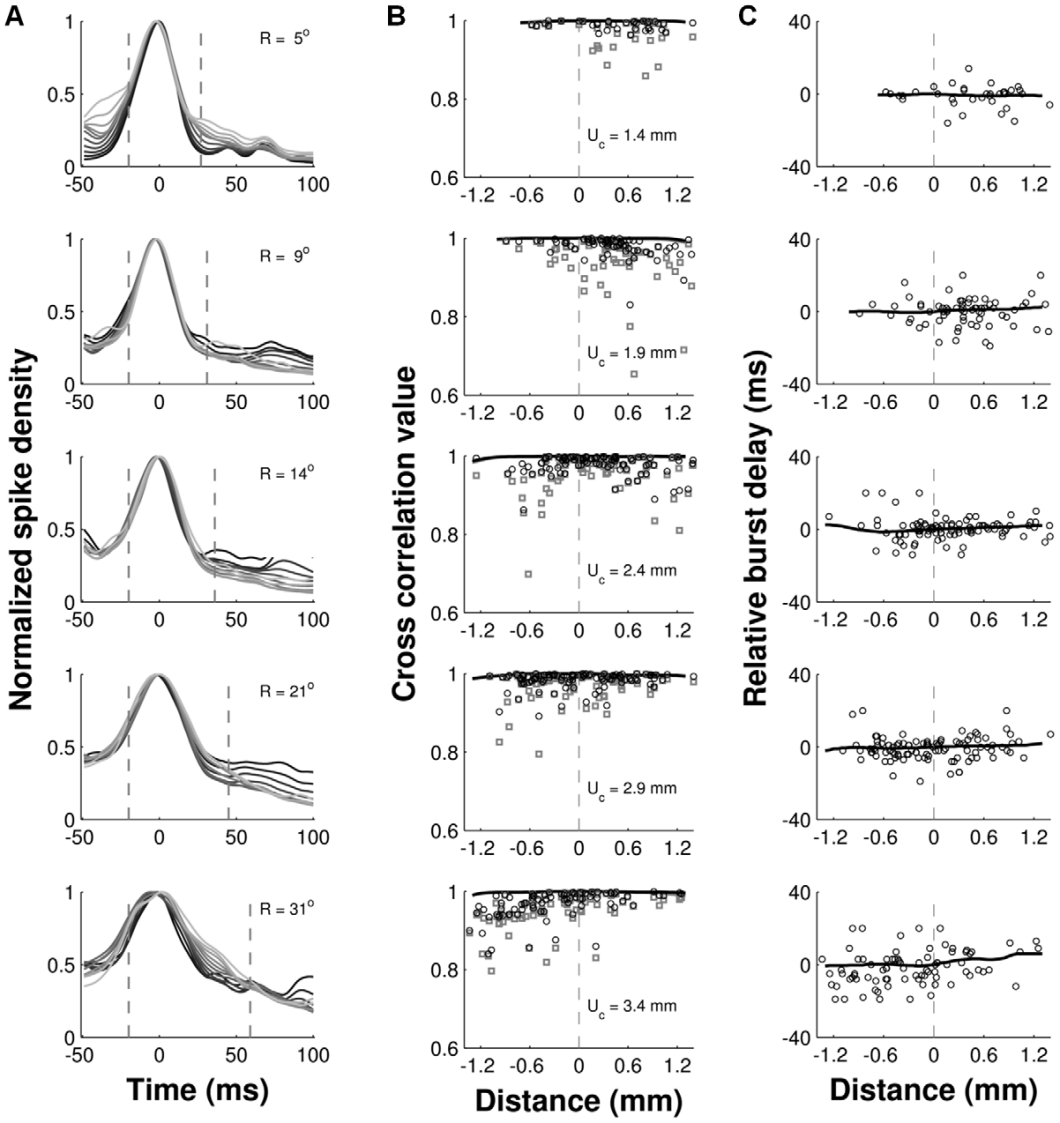
Recruited SC neurons synchronize their burst profiles. Temporal cross-correlation analysis of saccade-related burst activity for saccades of different amplitudes. **A**) Site-dependent burst profiles (from Fig. 9C) normalized with respect to their peak. Burst profiles in each panel are drawn at 0.2 mm intervals, starting 1 mm rostral (dark) to the center of the recruited population, and ending 1 mm caudal of it (bright). **B–C**) The central burst profile was taken from 20 ms before saccade onset until saccade offset (dashed lines in A) and cross-correlated with the population activity (solid lines) at different rostral-to-caudal distances from the center (negative values are more rostral, positive values are more caudal), and with the spike density functions of the individual cells (open symbols; from top to bottom: n = 35, n = 71, n = 99, n = 104, and n = 85). Positive delays in C indicate that the burst at a given location occurs later than at the center of the active population. Gray lines and symbols in B are the cross-correlation values obtained at time lag zero. Black lines and symbols are the correlations obtained at the optimal delay (i.e., peak of the cross-correlation function). U_c_: motor map coordinates of the center of the population taken along the horizontal meridian.

**Figure 12:**
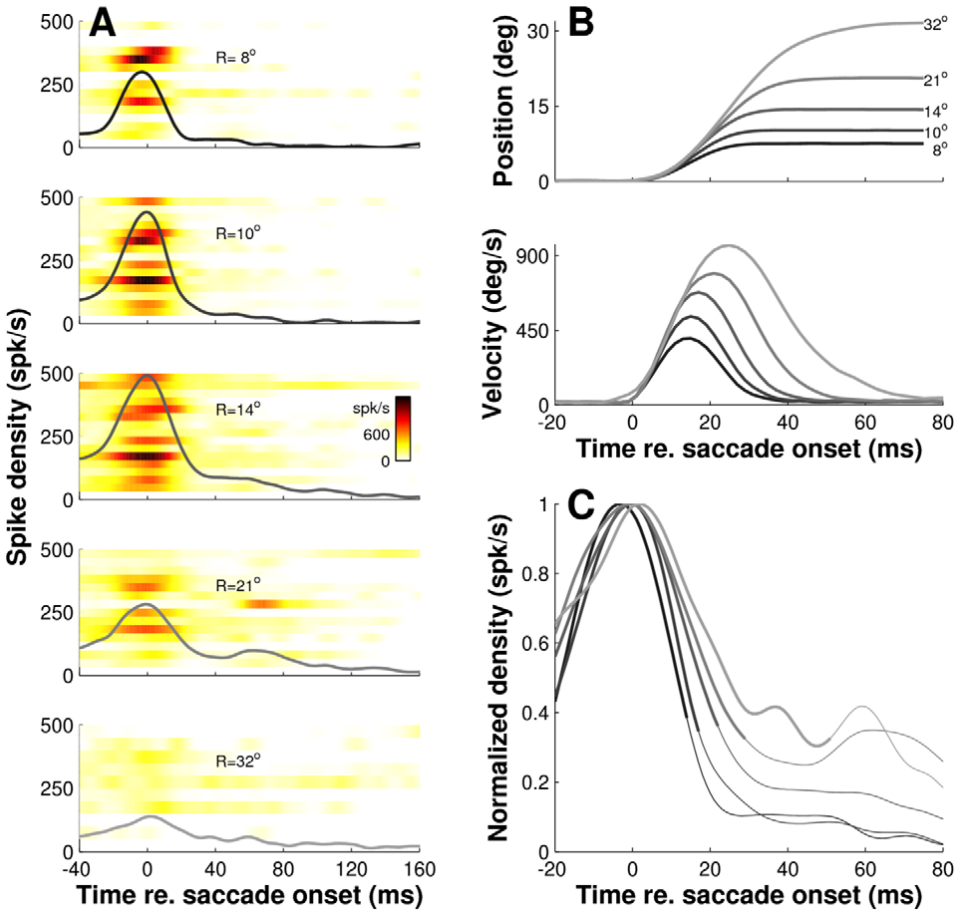
Burst shapes depend on planned movement. Shown are the saccade-related discharges of 16 neurons located in a small, central region of the SC for five different saccade amplitudes. Preferred amplitudes of the cells ranged from 13 to 15°. **A**) Instantaneous firing rates of the individual cells (color code) averaged across trials with the population average superimposed. Insets: schematic drawing of population activity relative to the recording sites. **B**) Eye position traces and eye velocity profiles of the corresponding eye movements show the main sequence behavior. **C**) Averaged spike density functions for the different saccade amplitudes normalized to their peak. Note the systematic increases in burst duration and skewness as saccade amplitude increases from 8 to 32 degrees, indicating that the shape parameters of the burst depend on the actual saccade in which the neurons participate, rather than on their topographic location in the motor map.

If the SC acts as an optimal controller, one would expect that cells in the recruited population synchronize their burst profiles because this would ensure that the net SC movement command specifies a straight trajectory with optimal horizontal and vertical velocity components. The example cells in Figs. 7 and 8 suggest that the burst shape of individual SC cells is indeed tuned such that all cells in the recruited population become synchronized for the saccade in which they participate. To determine whether burst synchronization in the recruited population is actually found across the SC, we studied the spatial-temporal burst dynamics of all recorded cells in the motor map for a range of different saccade amplitudes (Fig. 9) and directions (Fig. 10), cross-correlated the burst shapes of all recruited cells to determine their similarity (Fig. 11), and analyzed the burst shapes of a fixed set of neighbouring neurons for different saccade vectors (Fig. 12).

We first examined the temporal discharge profiles of saccade-related neurons along the rostral-to-caudal extent of the SC for saccades of five particular amplitudes. Because we applied the rotation algorithm (Methods), cells having different preferred directions could all be pooled.

Figure 9 shows this analysis for saccade amplitudes ranging from 5 degrees (top panels) to 31 degrees (bottom panels). Saccade directions always corresponded with the direction of the cells' preferred movement (Methods). The different plots in Fig. 9 thus provide estimates of the rostral-to-caudal cross-sections through the center of the population. As in previous figures, we indexed the rostral-to-caudal location of each cell by the amplitude of its preferred vector. The color-codes in Fig. 9A reflect the averaged spike density of individual cells as a function of time relative to saccade onset and their location in the SC motor map. The associated mean eye movements are superimposed. We calculated the activity profiles in Fig. 9B from the raw data in Fig. 9A by averaging the spike-density functions of nearby cells according to a Gaussian weighting function (width σ = 0.25 mm) and sorting them according to their location in the motor map (Methods). Bin centers were chosen at 2.5 deg intervals in visual space. In Fig. 9C, the site-dependent spike density functions are collapse onto a single pair of axes, and bin centers were chosen at 0.2 mm intervals on the SC motor map. The latter produced spike density functions of the population activity at equally spaced distances from the center of the active population. To facilitate visual inspection, the hue of the individual spike density functions indicates the rostral (green) to caudal (cyan) location of the cells while the color saturation is proportional to the firing rate (i.e., traces become darker when activity increases).

Note that for a given saccade amplitude the burst profiles along the rostral-to-caudal extent of the SC appear to have very similar shapes, which change systematically as function of saccade amplitude (and duration). More specifically, peak firing rates in the recruited population decrease systematically with increasing saccade amplitude while burst duration and skewness increase with increasing saccade amplitude. The data thus demonstrate that not only the location of the recruited population changes systematically with saccade amplitude; also the dynamics of the burst activity within the active population changes systematically.

The observation that cells at different locations *within* the active population all have very similar burst dynamics is further illustrated in Fig. 10. As in Fig. 9, we plot the responses of cells as a function of their rostral-to-caudal location in the SC, but now for 14 degree amplitude saccades in five different directions, *ψ*, relative to their preferred vector. The plots in Fig. 10 thus provide rostral-to-caudal cross sections through the population for a 14 degree saccade at five different iso-direction lines. Iso-direction line *ψ* = 0 deg runs through the center of the active population, so the plots in the center row of Fig. 10 are equivalent to the ones in the center row of Fig. 9. The other iso-direction lines, however, characterize the temporal discharge profiles at different medial-lateral locations.

Note that the peak firing rates decrease systematically with increasing rostral-to-caudal and increasing medial-to-lateral distance from the center of the population activity while the shape of the temporal burst profiles remains remarkably similar. In fact, it is not difficult to see that practically all discharge profiles in Fig. 10 are approximately scaled versions of each other. Our findings thus demonstrate that the temporal dynamics of burst activity is very similar throughout the population of recruited cells.

To quantify the temporal synchrony and shape similarity of the burst profiles at different locations within the active population, we performed a series of temporal cross-correlation analyses (see Methods, for details). As shown in Fig. 11A, we first normalized the site-dependent spike density functions from Fig. 9C with respect to their peak. For each saccade amplitude, we then cross-correlated the population activity at the center of the activated region of cells with the population activity at different rostral-to-caudal distances from the center (solid lines), and with the activity of the individual cells (open symbols).

Note that for each of the five movement amplitudes the normalized population responses fall on top of each other (Fig. 11A). Accordingly, the cross-correlation analyses performed on the population data (solid lines) produced correlation values at lag zero that were close to one (Fig. 11B), and optimal delays that were close to zero (Fig. 11C). These results thus indicate that the response profiles are indeed synchronized, scaled versions of each other. Even at the level of the individual cells it is observed that the cross-correlation values at lag zero are very high (gray squares; typically r(τ = 0)>0.8). For about 50–70% of the cells, the cross-correlation values at the optimal delay (black circles) were significantly higher than at lag zero (Fig. 11B), but the burst delays of the individual cells were not systematically related to their rostral-to-caudal location within the recruited population (Fig. 11C). The same pattern of results was obtained along the medial-lateral dimension of the population (data not shown). The cross-correlation values themselves were of course influenced to some extent by the width of the Gaussian smoothing kernel (here,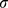 = 5 ms; Methods), but the resulting optimal delays were not. When we repeated the analysis with different kernel widths (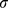 in the range of 2 to 10 ms), they were virtually identical. The robust analysis of relative burst delays in Fig. 11 thus demonstrates that there is no systematic spread of activity, neither in the rostral-to-caudal direction [31], [46] (which would produce a systematic increase of the lag), nor from the center towards the periphery of the population [27], [47] (which would produce a V-shape pattern).

Note, however, that the response profiles for saccades of different amplitudes are clearly different. I.e., burst profiles within the active population become more and more skewed as saccade amplitude and duration increases. The interesting question then arises whether these systematic changes in burst shape are also reflected in the population activity at a given location in the SC when the cells at that location participate in the generation of saccades of different amplitudes (and durations). The results of Figs. 7 and 8, in which we analyzed the responses of two example cells for small versus large saccades, would indeed suggest such changes.

To address this question, we selected in Fig. 12 a cluster of 16 neurons located in the central region of the SC. Preferred amplitudes of these cells were closely matched, ranging between 13–15 degrees. From the responses of the individual cells (color code in Fig. 12A) we first calculated the mean population response at that location for five different saccade amplitudes (solid lines in Fig. 12A), and we then normalized the resulting response profiles with respect to their peaks (Fig. 12C). Note that there are systematic increases in burst duration and skewness as the saccade amplitude increases from 8 to 32 degrees. Also note the main-sequence behavior in the corresponding eye movement traces of Fig. 12B.

## Discussion

Our data reveal a remarkably systematic organization of burst properties along the rostral-to-caudal extent of the SC motor map. These novel findings support our theoretical extension of the linear ensemble-coding scheme which explains how a gradient of burst properties in the deeper layers of the SC could underlie the nonlinear kinematics of saccades, their straight trajectories in oblique directions, and the skewed shape of their velocity profiles [37].

In support of our theoretical prediction, we found a clear rostral-to-caudal gradient in the burst profiles of SC cells when they are recruited for their preferred saccade: rostral cells have nearly symmetrical temporal burst profiles of short duration and high peak-firing rates, whereas caudal cells have highly skewed bursts of longer duration and much lower peak-firing rates. The number of spikes in these bursts, however, is constant across the map, at about 18–20 spikes on average. Interestingly, our findings also show that all cells in the recruited population synchronize their bursts, as they start, end, and reach their peak nearly simultaneously. Thus, the shape of the motor burst of a given cell is not determined by its location within the motor map, but by the saccade for which it is recruited. This precisely tuned organization of the population supports the notion of an optimal motor-control principle embedded in the SC motor map.

### Dynamic population code

We previously showed that a simple linear ensemble-coding model of the SC motor map could fully account for the nonlinear properties of saccades [33]. By driving a linear model of the brainstem with actual recorded spike trains from a large population of SC neurons widely distributed across the motor map we obtained realistic, straight saccades with the correct kinematic properties (Video S1). The assumptions of this linear ensemble-coding model (Fig. 1C) are the following:

- In line with neuroanatomical data [48]–[51], each spike of each recruited cell contributes a fixed movement (‘spike vector’) to the saccade, 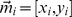, which is only determined by the cell's location, 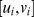, in the motor map according to the efferent mapping function:
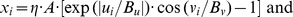


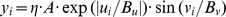
with 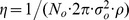 a scaling constant that is the same for all cells. The fixed parameters [A, B_u_, B_v_] = [3.0 deg, 1.4 mm, 1.8 mm/rad] describe the anisotropic and inhomogeneous geometry of the motor map [26], [28]; ρ is the cell density, N_0_ the maximum number of spikes in the burst of SC cells, and σ_0_ the width of the Gaussian population of recruited cells. Our recordings indicate that both N_0_ and σ_0_ are constant across the map (N_0_≈18–20 spikes, and σ_0_≈0.5 mm). Thus, if cell density does not vary along the motor map either, our current results imply that the total number of collicular spikes associated with saccades would be constant too.
- Linear summation of all spike vectors determines the desired two-dimensional saccade trajectory:
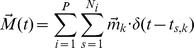
with *P* the number of active cells in the population, and *N_i_* the number of spikes generated by cell *i*. This crucial assumption states that the SC output is a dynamic desired eye-displacement signal that grows from zero to the final saccade amplitude in synchrony with the spike trains (i.e., the spatial-temporal integration stage in Fig. 1C). This assumption differs radically from alternative models, which assume that the SC population encodes the goal, not the motor command, for the upcoming saccade by weighted averaging of individual cell contributions [43]–[45], [52]. In weighted-averaging schemes, the saccade amplitude is specified virtually instantaneously and does not develop during the course of the saccade-related burst, i.e. 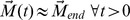 (Fig. 1B). These alternative models therefore provide no explanation as to why caudal cells would have to fire at much lower peak rates than rostral cells (the difference is, on average, about 50%), or why the shape of the motor burst of a given cell would have to depend systematically on the saccade for which it is recruited.
- The brainstem saccade generator decomposes the dynamic vectorial SC signal into its horizontal and vertical eye-velocity components, but is described by a *linear* input-output characteristic (Fig. 1C). In virtually all previous models (see [53], for review) the brainstem saccade generator embodies the nonlinear kinematics of saccades through a nonlinear, saturating characteristic. The saturation was therefore not explained, but added to the model to fit eye velocity profiles. By removing this nonlinearity from our model we could demonstrate that the SC output contains the information needed to generate the main-sequence properties and component stretching without additional assumptions.
- The motor neurons of the extra-ocular eye muscles receive eye-velocity commands from the brainstem pulse generator as well as eye-position step signals from parallel neural-integrator pathways (Fig. 1C). As in previous models [11]–[13] this so-called pulse-step innervation acts an inverse model of the oculomotor plant (which has a linear low-pass filter characteristic) to fully compensate for its sluggish dynamics.

Note that our scheme does not make any prior assumptions about the activity patterns of individual cells in the SC motor map. For example, the finding that the number of spikes in the burst is invariant for fast and extremely slow eye movements [54], and that it is invariant across the motor map (Fig. 4A) are not properties of the model, but appear to be properties of the motor map. The same holds for our current findings that the peak firing rate of cells, their burst duration and their burst skewness all vary in a systematic way with their location in the motor map. Finally, that all cells within the population are synchronized during the saccade, and that the burst properties are determined by the saccade in which the cell participates, rather than by its location in the map, is not a model assumption either. Yet, all these features taken together fully explain how the SC population could encode the nonlinear kinematics, and at the same time generate straight saccades in all directions.

The nonlinear behavior of saccade kinematics is due to two opposing factors: (i) peak firing rates in the caudal SC are lower than in the rostral SC, but (ii) each spike in the caudal SC has a much larger impact on the brainstem than a rostral cell, due to the exponentially growing efferent mapping function. Thus, although spike firing-rates decrease in the caudal SC, the increase in eye displacement provided by each spike is much larger at these locations. Straight saccades result from synchronization of burst profiles, especially along the medial-lateral dimension of the SC (Fig. 10). This synchronization ensures that the horizontal and vertical velocity commands are scaled versions of each other as is required for producing straight oblique saccades. Our findings thus strongly support the idea that the SC motor map acts as a nonlinear vectorial pulse generator.

Because the saccade results from the linear contribution of a large ensemble of recruited cells, our linear ensemble-coding model does not necessarily predict that each individual cell should encode the details of the saccade kinematics. Nevertheless, the saccade kinematics are to a large extent reflected at the level of single cells, as the shape of the saccade-related burst follows a similar skewness relationship with burst duration as the saccade velocity profile to saccade duration (Fig. 6). In addition, the burst shape of any individual cell is to a large extent determined by the saccade metrics, and can thus vary substantially between small and large saccades into its movement field (Figs. 7, 8 and 12). Interestingly, these features are predicted by the notion that saccade-related SC neurons have dynamic movement fields which determine the dynamic relationship between the activity of individual cells and the ensuing eye displacement as a function of the saccade metrics [33]. This concept also predicts that for movements to a single visual target the recruited cells act together as a ‘common source’ by synchronizing their burst profiles. This behavior is indeed observed (Figs. 9–11).

Taken together, our findings provide strong support for the argument that the nonlinear saccade kinematics are not due to a passive saturation of brainstem burst neurons, e.g., as a result of neural fatigue, but reflect a deliberate design property within the saccadic system to produce the main-sequence characteristics.

### Optimal control

We believe that such a strategy aims to optimally cope (in a statistical sense) with conflicting constraints that impede the fovea from getting as fast and as accurately as possible on a peripheral target of interest. Several constraints may be identified within the system: neural noise, considerable processing delays, and the highly inhomogenous organization of the retina that introduces considerable uncertainty about stimulus locations within the visual periphery. Indeed, theoretical studies on optimization have provided an elegant explanation for different features of saccadic behavior, such as the tendency to systematically undershoot visual targets [55], but also the main-sequence nonlinearity [4]–[7]. In these studies, the noise is assumed to be multiplicative, i.e. signal-dependent. To optimize such a system for speed and accuracy, the control signal should obey the nonlinear main sequence, and at the same time employ an undershooting strategy. Our data show for the first time that SC cells indeed possess multiplicative, signal-dependent noise (Figs. 2 and 3), and that this property is invariant across the motor map (Table 1).

Since the coefficient of variation is, on average, constant across the map, it follows from our spike-vector summation theory that the variance in the resulting displacement vector, 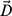, as function of desired amplitude and direction is given by:
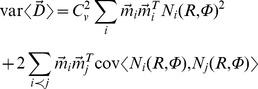
Where *N_i_*(*R,Ф*) is the mean number of spikes fired by cell *i*, 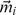 its spike-vector contribution, and *C_v_* the coefficient of variation (population average). Clearly, the covariances in this equation cannot be determined from our single-unit recordings, so we cannot be 100% sure that the net collicular output has multiplicative, signal-dependent noise. Simulations with a uniform motor map (i.e., fixed cell density, 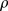, and location-invariant widths *σ_mf_* and heights *N_pref_* of the Gaussian movement fields) showed, however, that both correlated and uncorrelated cell activities produce elliptical endpoint distributions (e.g., [56]) with standard deviations that increase linearly with eccentricity (not shown).

The optimal control models described above all dealt with the generation of horizontal saccades. However, in two dimensions the fastest response should also follow a straight line; thus an extended optimal control theory would predict straight oblique saccades [8]. In alternative models, in which the brainstem is driven by separate horizontal and vertical nonlinear pulse generators, the programming of straight oblique saccades is highly nontrivial as it requires a tedious scheme of cross-coupling between the horizontal and vertical systems [21]–[23]. The problem is even more complex when considering head-free gaze shifts [57]–[59], because a fixed cross-coupling scheme will no longer work when the eye-head coupling varies considerably from trial to trial. However, if the nonlinear burst generator resides in the SC motor map, straight saccades (and head-free gaze shifts) become an emerging property of the system, requiring no further cross-coupling than a simple linear horizontal/vertical decomposition (i.e. sine and cosine projection) of the vectorial gaze-shift command.

### Bursting mechanisms

Our simple linear ensemble-coding model is still incomplete. It accounts for the generation of saccadic eye movements once the spatial-temporal distribution of SC activity is known, but it does not explain how the tuned burst patterns come about in the first place (an omission in most models of the saccadic system). In principle, the saccade-related burst of SC neurons could be derived from two alternative mechanisms. First, the type of input signal, such as that from the frontal eye fields (FEF), might impose the spatial-temporal pattern of excitation [60]. However, this explanation is neither attractive nor plausible; it merely shifts the problem to a different area of the brain, and there exists (at least to our knowledge) no evidence that the FEF is involved in the dynamic, online control of saccade trajectories under normal conditions. Second, the burst might arise from intrinsic membrane properties of saccade-related SC neurons [61], or from properties of the local circuits within the SC motor map. Recent *in vitro* experiments indeed suggest that the synchronous bursting command observed in our data could result from a local excitatory network, in combination with NMDA receptor activation in the deeper layers of the SC [62]–[64]. These *in vitro* experiments also demonstrated a strong nonlinear signal amplification process in the SC, which is interesting because it might account for the nonlinearity (i.e., vector averaging) of responses obtained with certain types of electrical double stimulation [65]–[67].

In theory, feedback from the brainstem saccade generator could also contribute to the shaping of the burst dynamics. For example, Van Opstal and Kappen [68] suggested that a linear model of the brainstem together with weighted feedback projections to the SC motor map reproduces straight saccades with the correct kinematics. However, in their scheme caudal cells receive the weakest feedback, and the model therefore predicts a strongly asymmetric distribution of burst durations and skewness within the recruited population. This is clearly not observed in our data (Figs. 9–11). Moreover, previous perturbation studies have shown that activity in the SC is also not consistent with other types of feedback models, as the SC activity does not encode dynamic motor error [32], [54], [69]–[74].

### Conclusion

Further research is needed to elucidate the mechanisms that shape the spatial-temporal firing patterns during saccades, and the behavior of the SC population in more complex motor behaviors, like during head-free gaze shifts, curved double-step saccades, or electrical microstimulation. Nevertheless, the burst properties reported in this study strongly support the idea that the deeper layers of the SC act as an optimal controller: the systematic organization of peak firing rates and burst durations as function of saccade amplitude along the motor map, the synchronous change in firing rate of recruited cells in the population, and the shaping of the temporal burst profile of a given cell with the currently planned saccade, all contribute to the generation of straight eye-movement trajectories with optimal kinematics.

## Methods

### Experimental procedures

We collected data from four rhesus monkeys (*macaca mulatta*) that were trained to follow a small visual target with saccadic eye movements. The setup, surgical procedures and behavioural paradigms have been described elsewhere [33], [54], [71]. In short, the animals were seated in a primate chair facing a spherical array of light-emitting diodes (LEDs) in an otherwise completely dark room. The head was restrained, and movements of the eye were measured with the double-magnetic induction technique [75], [76]. Single-cell recordings were made through a recording cylinder using tungsten microelectrodes that were advanced into the SC with the use of a hydraulic stepping motor.

We recorded activity of 146 saccade-related neurons that were found in the intermediate and deep layers of the SC (about 0.5–3 mm below the dorsal surface). Cells were considered saccade-related if they showed an increase in firing rate around the onset of saccades towards a particular region of the visual field. All of these cells were studied with a standard saccade task in which the animal made saccades from an initial fixation LED to a peripheral target LED which was presented for 500 ms. The movement field of each neuron was determined by eliciting saccades to targets inside and neighboring the response field (‘movement field scan’). In addition, saccades were evoked to a fixed series of targets across the visual field (*R* between 2–35 deg, *Φ*∈[0,30,…,360] deg; ‘rose scan’). Eye movement data, spike data, and movement field parameters of all 146 recording sites were stored in a database for further processing. The file contained data from a total of 32,147 trials.

#### Ethics statement

To ensure the animals' health and welfare, their general appearance was monitored on a daily basis and recorded in a welfare diary, along with their daily food and fluid intake. Surgery was performed under general anesthesia, and postoperative pain treatment was applied for at least three consecutive days. All experimental procedures were in accordance with the European Communities Council Directive of November 24, 1986 (86/609/EEC), and were reviewed and approved by the local university ethics committee.

### Data analysis

Saccades were detected off-line on the basis of the calibrated eye-position signals using custom software (see [71] for details). Subsequent analysis was done in Matlab 7.9 (version R2009b).

Single-cell activity was displayed in spike rasters and peri-stimulus time histograms (PSTHs) that were aligned on specific events such as target onset and the onset of a saccade. Spike trains from individual trials were represented as a sequence of δ pulses at the time of spike occurrence, 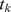 (1 ms resolution): 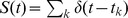. Spike density functions, 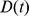, were calculated from these raw spike trains by convolving 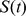 with a Gaussian smoothing kernel that had a default width of 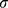 = 5 ms and a height of 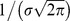 spk/s, but analyses were also repeated for different kernel widths 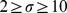 (see text).

#### Burst parameters

To quantify the burst properties of saccade-related neurons, we considered spikes that occurred in a time window ranging from 20 ms before saccade onset until 20 ms before saccade offset. We adopted a fixed lead-time of 20 ms because this value corresponds with the typical latency of saccades evoked by electrical stimulation at the recording sites. The number of spikes in the burst, *N_s_*, was counted within this variable time window. Mean firing rates, *F_m_*, were defined as the number of spikes in the burst divided by saccade duration. Peak firing rates, *F_p_*, and the time to peak activity, *T_p_*, were determined from averaged spike density functions, where data from individual trials were first aligned with saccade onset. Negative values of *T_p_* indicate that the peak of the burst precedes saccade onset. Burst skewness, 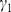, quantified the skewness of the raw PSTHs over the spike-analysis window, aligned with saccade onset, and was calculated directly as the third moment from the distribution of spike occurrences relative to saccade onset:
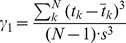
(1)where 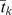 is the mean, 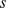 the standard deviation, and 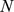 the number of spikes in the burst. Negative values indicate burst profiles that are skewed left and positive values indicate burst profiles that are skewed right. By skewed left, we mean that the left tail is longer than the right tail. Similarly, skewed right means that the right tail is longer than the left tail.

#### Movement fields

For each cell, we obtained a quantitative description of its movement field from a series of saccade responses towards targets at various locations within and beyond the response field of the cell (see, e.g., Fig. 2, for illustration). We used the movement field model of Ottes et al. [28] to account for the complex-logarithmic nature of the SC motor map. First, we mapped the polar coordinates *[R,Φ]* of each saccade vector in visual space onto Carthesian coordinates *[u,v]* of the SC motor map using the afferent mapping function *[R,Φ]→[u,v]* :
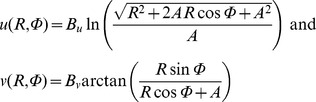
(2)where *A* = 1.4 mm, *B_u_* = 1.8 mm/rad, and *B_v_* = 3.0 deg are fixed parameters of the motor map model. Then, using the number of spikes in the burst as a measure of burst magnitude, we quantified the vector-dependent activity of the cell by assuming a two-dimensional Gaussian activation profile in this motor map:
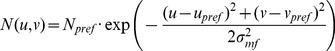
(3)Parameters *[u_pref_,v_pref_]* (in mm) define the center of the Gaussian in Cartesian motor map coordinates, which corresponds with the point image of the cell's preferred vector *[R_pref_,Φ_pref_]* in visual space. *σ_mf_* (in mm) is the width of the cell's Gaussian activation profile. *N_pref_* is a measure of the peak activity of the cell associated with its preferred vector. The four free parameters, *u_pref_*, *v_pref_*, *σ_mf_* and *N_pref_*, were estimated with a nonlinear least-squares fit procedure (Nelder-Mead simplex method; see [33], for details and results).

#### Spike-count variability

The trial-to-trial variability in spike counts, *N_s_*, was characterized by the ratio of the standard deviation to the mean:
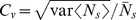
(4)This quantity is often referred to as the coefficient of variation. In our analysis, we determined 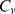 from the slope of linear regression lines fitted to 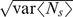 as a function of 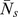 (see Fig. 2D, for an example). For each cell, we obtained this relation by selecting clusters of closely matching saccade responses into the movement field of the cell. We used data-clustering routines implemented in Matlab to select non-overlapping clusters that contained at least five responses for which the Euclidean distance between individual saccade vectors on the SC motor map was less than 1⋅*σ_mf_* (in mm). We used Cartesian motor map coordinates, *[u,v]*, because they conveniently account for the amplitude asymmetry of movement fields in visual space. The population analysis (Fig. 3) included only cells having at least five response clusters in which the range of mean spike counts spanned at least 30% of the peak activity (i.e., max(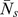)−min(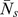)>0.3⋅*N_pref_* ).

Although the range of saccade vectors in each cluster was small, there is some concern that the trial-to-trial variability in saccade amplitude and direction leads to an overestimate of the neural noise at locations within the movement field where its gradient is steepest. To estimate the true intrinsic noise of each neuron, we therefore substituted, in Eq. 4, the spike-count variance 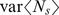 with the residual variance 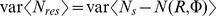 because this latter measure corrects for spike-count variability explained by its movement field. 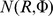 for each saccade was obtained from Eq. 3. Without this correction, the overall result across our population of cells was not significantly different. But since it produced overestimates of the variance in some cells, we decided to use the correction for all neurons for consistency.

#### Responses into movement field center

Figures 4–6 quantify the burst properties of SC cells for saccades into the center of their movement field. For each cell included in these analyses (n = 103), we selected saccade trials in the following way. First, we selected all trials in which the saccade vector ended within 0.2⋅*σ_mf_* from the center of the movement field. If this range included less than 5 trials, the selection criterion was gradually widened (step size 0.01⋅*σ_mf_*) until at least 5 responses were obtained, or until the limit exceeded 0.5⋅*σ_mf_*. This selection procedure ensured that we only included those trials in which the saccade vector was closest to the movement field center (typically within 0.4⋅*σ_mf_*). Cells for which we obtained less than 5 saccades within 0.5⋅*σ_mf_* from the center (n = 43) were excluded from these particular analyses.

#### Population activity

To generate plots of SC population activity (Figs. 9–10), we first mapped the preferred vector of each neuron onto the right horizontal meridian. This involved a clockwise rotation of the preferred vector *[R_pref_,Φ_pref_]* by *Φ_pref_* degrees. The actual eye movements were re-expressed in this new frame of reference too by rotating each saccade vector by the same amount. In this way, all saccades in the direction of the preferred vector of each cell had a direction 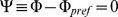 deg.

From our database of cell responses we then selected all trials in which the saccades closely matched our ‘target’ vector (i.e., the movement for which we wanted to compute the population activity). The criterion for matching the responses across cells and trials was defined in motor map coordinates. More specifically, the Euclidean distance, *d*, between the target vector *[u_t_,v_t_]* and the (rotated) movement vector *[u_i_,v_i_]* had to be less than 0.3 mm.

For each cell, we then calculated a weighted average of its spike density functions across trials, where the weight of each trial, *i*, depended on *d* so that responses with the best matching movements had the largest weights:
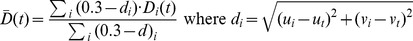
(5)Finally, the population activity at each rostral-to-caudal location in the motor map, *u*, was calculated by averaging the spike density functions of nearby cells according to a Gaussian weighting function:
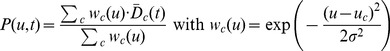
(6)where σ = 0.25 mm is the width of the spatial smoothing kernel and *u_c_* the rostral-to-caudal location of each cell *c* taken along the horizontal meridian of the motor map.

#### Cross-correlation analysis

To test whether the burst profiles of cells at different locations within the active cell population are synchronized, scaled versions of each other, we cross-correlated the temporal firing pattern at the center of the recruited population with the firing patterns at different rostral-to-caudal distances from the center (Fig. 11). In this analysis, we took the population activity at the center of the recruited population from 20 ms before saccade onset until saccade offset. This center burst-profile, 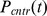, was then shifted in time and correlated either with single-unit activity or with population activity at a different site, 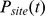. Cross-correlation values at each time delay, τ, were obtained from:
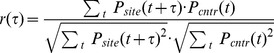
(7)where *t* runs from 20 ms before saccade onset until saccade offset. The resulting cross-correlation functions, *r(τ)*, are normalized so that the auto-correlations at *τ* = 0 are equal to 1.

Peaks of the cross-correlation functions were used to determine the relative timing of burst activity within the recruited cell population. Positive delays indicate that the burst activity at a certain site occurs later in time compared with the burst activity at the center, while negative delays indicate that burst activity at that location precedes the burst activity at the center.

We restricted our analyses to population activity within 1.25 mm from the center of the recruited population (i.e., ∼2.5 times the width *σ_mf_* of the average movement field) because the burst activity at larger distances rapidly dropped to zero. For individual cells, we also required that the number of spikes in their burst exceeded 5% of the number of spikes that they fired for their own preferred vector (i.e., *N_s_*>0.05⋅*N_pref_*).

## Supporting Information

Video S1 Reconstruction of nine different saccades from measured SC activity patterns using our linear two-dimensional model of the SC – brainstem saccade generator (c.f., Fig. 1C). The video shows the activity patterns in the left, contralateral SC (top left), the two-dimensional eye movement trajectories (bottom left), the horizontal and vertical eye position traces (top and middle right), and the vectorial eye velocity profiles (bottom right). Amplitude and direction of the subsequent movements is indicated by the [*R*,Φ] coordinates (bottom left). Note that the reconstructions (green) reproduced the straight trajectories, component stretching, and nonlinear kinematics of the measured saccades (blue) quite well, even though none of these properties were built into the model. Details of the reconstruction procedure have been described elsewhere [33]. In short, we first estimated the dynamic SC activity associated with a particular saccade vector from the cells' responses recorded during saccades of that particular amplitude and direction. Towards that end, we mapped each cell's spikes directly onto its location in the SC motor map, and from the measured spike events at each recording site we then calculated spatially smoothed maps of the instantaneous firing rates. The resulting estimates of the SC firing patterns in space and time were subsequently decomposed into dynamic horizontal and vertical movement commands using our spike vector summation model, which assumes that each spike from each SC neuron adds a tiny, site-specific contribution to the horizontal and vertical movement commands. The brainstem circuit for the horizontal and vertical eye-movement components was modeled by two independent linear feedback systems (c.f., Fig. 1C). The three model parameters (a fixed delay between SC and brainstem activation, the feed forward gain of the pulse generators, and a fixed delay in the local feedback loops; fixed for all reconstructions) were determined by fitting the reconstructions to the measured saccades.
(MPG)
